# Considering health equity when moving from evidence-based guideline recommendations to implementation: a case study from an upper-middle income country on the GRADE approach

**DOI:** 10.1093/heapol/czx126

**Published:** 2017-10-03

**Authors:** Javier Eslava-Schmalbach, Paola Mosquera, Juan Pablo Alzate, Kevin Pottie, Vivian Welch, Elie A Akl, Janet Jull, Eddy Lang, Srinivasa Vittal Katikireddi, Rachel Morton, Lehana Thabane, Bev Shea, Airton T Stein, Jasvinder Singh, Ivan D Florez, Gordon Guyatt, Holger Schünemann, Peter Tugwell

**Affiliations:** 1 Equity-in-Health Group, Faculty of Medicine, Hospital Universitario Nacional de Colombia, Universidad Nacional de Colombia, Cra 30 45-03, University Campus, Bogota, Colombia; 2 Technology Development Centre, Colombian Society of Anesthesiology and Resuscitation (S.C.A.R.E.), Carrera 15A 120-74, Bogota, Colombia; 3 Epidemiology and Global Health, Department of Public Health and Clinical Medicine, Umeå University, 901 87 Umeå, Sweden; 4 Equity-in-Health Group, Clinical Research Institute, Faculty of Medicine, Universidad Nacional de Colombia, Bogotá, Colombia; 5 Bruyère Research Institute, University of Ottawa, 85 Primrose Avenue, Room 312, Ottawa, ON K1R 7G5, Canada; 6 School of Epidemiology, Public Health and Preventive Medicine, University of Ottawa, Ottawa, ON, Canada; 7 Ottawa Hospital Research Institute, Ottawa, ON, Canada; 8 Department of Clinical Epidemiology and Biostatistics, McMaster University, Hamilton, ON, Canada; 9 Clinical Epidemiology Unit, Department of Internal Medicine, American University of Beirut Medical Center, Beirut 1107, 2020 Lebanon; 10 Department of Emergency Medicine, Foothills Medical Centre, Calgary, AB, Canada; 11 MRC/CSO Social and Public Health Sciences Unit, University of Glasgow, Glasgow G2 3QB, UK; 12 Sydney School of Public Health, The University of Sydney, Sydney 2006 NSW, Australia; 13 McMaster University, Hamilton, ON, Canada; 14 Clinical Epidemiology Program, Ottawa Hospital Research Institute, Ottawa, ON, Canada; 15 Public Health Ufcspa, Ulbra, HTA of Conceicao Hospital, Porto Alegre, Brazil; 16 Medicine Service, Birmingham VA Medical Center, Birmingham, AL, USA; 17 Division of Epidemiology, Department of Medicine, School of Medicine, School of Public Health, University of Alabama at Birmingham (UAB), Birmingham, AL, USA; 18 Department of Orthopedic Surgery, Mayo Clinic, College of Medicine, Rochester, MN, USA; 19 Department of Pediatrics, Universidad de Antioquia, Medellin, Colombia; 20 Department of Clinical Epidemiology and Biostatistics, McMaster University, Hamilton, ON, Canada; 21 Department of Medicine, McMaster University, Hamilton, ON, Canada; 22 Clinical Epidemiology Program, Department of Medicine, University of Ottawa, Ottawa, Canada

**Keywords:** Health inequalities, clinical, practice guidelines, implementation, equity

## Abstract

The availability of evidence-based guidelines does not ensure their implementation and use in clinical practice or policy making. Inequities in health have been defined as those inequalities within or between populations that are avoidable, unnecessary and also unjust and unfair. Evidence-based clinical practice and public health guidelines (‘guidelines’) can be used to target health inequities experienced by disadvantaged populations, although guidelines may unintentionally increase health inequities. For this reason, there is a need for evidence-based clinical practice and public health guidelines to intentionally target health inequities experienced by disadvantaged populations. Current guideline development processes do not include steps for planned implementation of equity-focused guidelines. This article describes nine steps that provide guidance for consideration of equity during guideline implementation. A critical appraisal of the literature followed by a process to build expert consensus was undertaken to define how to include consideration of equity issues during the specific GRADE guideline development process. Using a case study from Colombia we describe nine steps that were used to implement equity-focused GRADE recommendations: (1) identification of disadvantaged groups, (2) quantification of current health inequities, (3) development of equity-sensitive recommendations, (4) identification of key actors for implementation of equity-focused recommendations, (5) identification of barriers and facilitators to the implementation of equity-focused recommendations, (6) development of an equity strategy to be included in the implementation plan, (7) assessment of resources and incentives, (8) development of a communication strategy to support an equity focus and (9) development of monitoring and evaluation strategies. This case study can be used as model for implementing clinical practice guidelines, taking into account equity issues during guideline development and implementation.

Key Messages

- For the implementation of guidelines that consider equity within the local context, a comprehensive approach is required.
- The implementation of an equity-focused guideline requires commitment from decision makers, inter-sectorial collaboration and involvement of the public.
- Effective communication, equity-focused monitoring and evaluation are crucial factors.
- Health and social systems, geographical and financial constraints may all challenge the implementation of equity-focused guidelines.

## Introduction

The World Health Organization (WHO) develops guidelines that are used by international organizations and endorses a rigorous process to ensure that guideline recommendations are based on the best available evidence. In addition, the WHO recognizes the importance of guideline implementation, and the use of standardized methods as well as the iterative adaptation of guidelines in implementation (Grimshaw *et al.* 1995; Wang *et al.* 2016). Despite the promotion of these rigorous methods, the availability of evidence-based guidelines does not ensure their implementation and use in clinical practice or policy making. There is recognition of the need for implementation tools and guidelines, as guideline developers may lack the resources to either incorporate implementation advice (Reyes *et al.* 2004; Gagliardi *et al.* 2011; Gagliardi and Brouwers 2012, 2015) or tailor guidelines to meet the unique needs of stakeholders (Brugha and Varvasovszky 2000).

Inequities in health have been defined as those inequalities that are avoidable, unnecessary and also unjust and unfair (Whitehead 1992). Although this definition has been critiqued (Norheim and Asada 2009) it provides a useful reference point to think about which inequities in health could be prevented, avoided or diminished. Evidence-based clinical practice and public health guidelines (henceforth ‘guidelines’) are mainly developed to improve quality of care in general, and they can also be used to reduce health inequities and improve care of disadvantaged population (Oxman *et al.* 2006; Dans *et al.* 2007; Mizen *et al.* 2012; Welch *et al.* 2017a). However, guidelines can unintentionally increase health inequities; e.g. when an intervention recommended within the guidelines ends up being of greater benefit to advantaged (lower-risk) groups than to disadvantaged (higher-risk) groups (Dans *et al.* 2007; Mizen *et al.* 2012). When interventions increase inequities, they are labeled ‘intervention-generated inequalities’ (IGIs) (Lorenc *et al.* 2013).

The concept of adopting an ‘equity lens’ for use with the development of guidelines has been introduced to denote a strategy that involves explicit consideration of equity aspects with the aims of ameliorating the inequities between the most and the least disadvantaged in any given health intervention (Nasser *et al.* 2013). This involves not only identifying interventions to reduce health inequities, but also identifying interventions likely to produce IGIs so that these adverse impacts can be mitigated. Modifying the guideline development process to incorporate an equity lens perspective includes exploring and anticipating the different effects of an intervention in disadvantaged populations, minimizing barriers to guideline implementation for disadvantaged populations and assessing the impact of the recommendations with disadvantaged populations (Dans *et al.* 2007). An equity proofing approach has been proposed, to occur before the implementation of policies and programmes, even if they are not equity-focused (Kelly *et al.* 2007).

GRADE (Grading of Recommendations Assessment, Development and Evaluation) is a well-developed formal process to rate the quality of scientific evidence in systematic reviews and to develop recommendations in guidelines that are as evidence-based as possible (GRADE Working Group 2004). Although the current GRADE guideline development process includes the GRADE Evidence to Decision (EtD) frameworks and related tools to consider the feasibility of guideline implementation (Alonso-Coello *et al.* 2016), it does not explicitly include steps for planned implementation. How guideline development panels plan the implementation of equity-focused guidelines have been found to lead to better outcomes for the population the guidelines are meant to benefit (Latham *et al.* 2012). The aim of planned guideline implementation is to minimize the risk of exacerbating existing health inequities and maximize health benefit for the whole population (Eslava-Schmalbach *et al.* 2016). We believe that the guideline development panel is well situated to plan guideline implementation. Such plans would be based on the evidence collected during the formulation of recommendations such as stakeholder values and preferences, as well on the guideline implementation process with access and feasibility.

**Figure 1.**
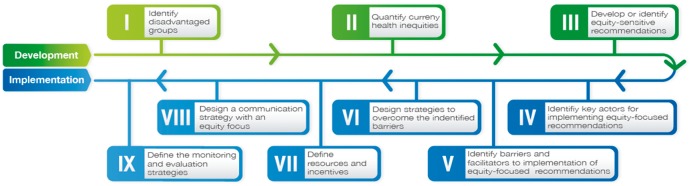
Steps in consideration of equity for guideline development and implementation

The purpose of this article is to describe nine steps that provide guidance for consideration of equity during guideline implementation. To illustrate the process, we use a Colombian case study on the development of the clinical practice guideline for the prevention, early detection and treatment of pregnancy, childbirth or puerperium complications (henceforth ‘CCase’) (Ministerio de Salud y Protección Social (Colombia) and Colciencias 2013b). The steps may be applied to three different types of guidelines: (1) equity focused, (2) those with a subset of recommendations targeting reduction of inequities and (3) guidelines developed for the general population without explicit focus on equity (see Table 1). The case study described here is a guideline with an equity recommendation (Type 2), and that was developed following the nine proposed steps. For guidelines without an explicit focus on equity (based on the focus of the guideline and the place in which it will be implemented), a minor or major adaptation to the nine steps may be needed. Steps IV–IX can be used as the pathways for the implementation of guidelines that have been developed without considering equity issues (Figure 1).

**Table 1.** Types of guidelines in which equity issues could be considered during implementation

Kind of evidence-based guidelines including equity issues	
a. Equity-focused guidelines designed to address identified equity issues as well as the effectiveness of proposed interventions	Example 1: The Philippines Dyslipidemia guideline (Philippine Heart Association 2008) in which the main objective was to develop valid and applicable dyslipidemia clinical practice guideline for Filipinos, with special consideration for existing health inequalities. They identified disadvantaged populations as those who live below the poverty threshold; cannot afford laboratory tests/exams and drug treatment; have limited access or no access to health care; or are undernourished
b. Guidelines developed for the general population and including a subset of recommendations for a subgroup and targeting the reduction of inequities or aimed at avoiding the exacerbation of existing inequities	Example 2: The Colombian Pregnancy guideline recommendation particular to health equity is as follows (Ministerio de Salud y Protección Social and Colciencias, 2013a):
	−The use of a balanced protein-energy supplement (i.e. proteins provide <25% of the total energy content) to reduce disparities in stillbirth for disadvantaged pregnant women, i.e. women who are malnourished or at risk of food insecurity who are at a higher risk of stillbirth (Good clinical practice recommendation).
	Example 3. The Colombian Sexually Transmitted Disease guideline (Ministerio de Salud y Protección Social and Colciencias, 2013b):
	−The use of a single dose (tinidazole 2 g + fluconazole 150 mg) for vaginal discharge treatment is suggested in disadvantaged women (poverty, sex workers etc.) (Strong recommendation in favour)
c. Public health guidelines or programmes developed for the general population	Example 4: The impact on health inequalities of a Glasgow neighbourhood renewal programme (Egan *et al.* 2016):
	− …investment in housing-led renewal in Glasgow was allocated according to population need and this led to modest reductions in social inequalities in health after 4 years (*P* = 0.036).

## Methodology

A critical review of the literature and a process to build expert consensus was undertaken to define how to include equity issues during the GRADE guideline development process. The methodology is based on a previously published theoretical approach, described elsewhere (Eslava-Schmalbach *et al.* 2016). In the instance of the Colombian case study discussed here, the nine steps are the result of using the previously developed theoretical approach. After engaging in a consensus-building process with the guideline’s developers, these nine steps were found to describe a focus on the guideline implementation process.

## Nine steps for equity-focused implementation

Guideline implementation should be considered and planned for during the process of guideline development. We summarize nine steps that were originally used to plan the implementation of two equity-focused guidelines (Ministerio de Salud y Protección Social (Colombia) and Colciencias 2013b, Ministerio de Salud y Protección Social (Colombia) and Colciencias 2013a). One of the guidelines was developed using the GRADE approach (Ministerio de Salud y Protección Social (Colombia) and Colciencias 2013a) and the other one was developed using an adaptation of a guideline development process used by NICE and the Center for Evidence Based Medicine of Oxford (Ministerio de Salud y Protección Social (Colombia) and Colciencias 2013b). The process of guideline development requires a stakeholder commitment to engage with and support guideline implementation, which may potentially include the same key actors identified in Steps IV–IX (Figure 1). The first three steps have been discussed in the GRADE Working Group series on Health Equity Methods (Akl *et al.* 2017; Pottie *et al.* 2017; Welch *et al.* 2017a,b) (Steps I–III):

I. ‘Identify disadvantaged groups’ who are affected by the issues the guideline is addressing, and may be disadvantaged in their access of the options recommended in the guideline (for details see Akl *et al.* 2017). In the definition of disadvantaged populations, it is relevant to consider populations that could be affected or influenced by more than one socially stratifying factor, and that may increase or mitigate the conditions of disadvantage they experience. For the specific CCase, pregnant women at a high risk of food insecurity and hunger were identified as a disadvantaged group, and the compounding of disadvantage across different factors such as low socio-economic status, gender and low-resource setting were considered to be important issues as well.
II. ‘Quantify current health inequities’ affecting the groups identified as disadvantaged (for details see Akl *et al.* 2017; Welch *et al.* 2017b). In the CCase, inequities in maternal mortality were quantified by highlighting the relationship between the unsatisfied basic needs index, the poverty index and standardized maternal mortality ratio (SMMR) (Sandoval-Vargas and Eslava-Schmalbach 2013). Highlighting the relationships between basic need and poverty index with the SMMR showed how inequities in SMMR are higher in places with more poverty and unsatisfied basic needs. For pregnant women these are proxy variables for being at risk of food insecurity and hunger.
III. ***‘***Develop or identify equity-sensitive recommendations’ (for details see Pottie *et al.* 2017). In the CCase, the guideline expert panel recommended as a good clinical practice recommendation the provision of balanced protein-energy supplementation to food insecure populations in general and malnourished pregnant women in particular, to reduce disparities in stillbirth rates found to exist for disadvantaged women.

The following Steps (IV–IX) follow from and build on these three steps:

### IV. Identify key actors for implementing equity-focused recommendations

The guideline panel first identifies key actors to be involved in the implementation of equity-focused recommendations. An actor mapping process is helpful to identify key actors within and outside the health sector that can facilitate the implementation of equity-focused recommendations (e.g. policy makers, health care providers, third party payers, patients, human rights defenders). Ideally, the actor mapping process should be initiated early in the guideline development process and inclusive of representatives of identified disadvantaged groups. To facilitate a successful guideline implementation process, the guideline panel must define specific responsibilities for each actor and sector involved in guideline implementation. Ideally, key actors should be involved throughout the entire guideline development process, from the guideline topic selection and scope definition through to implementation planning (Brugha and Varvasovszky 2000).

In the CCase, policy makers in health planning, implementation and evaluation (Ministry of Health and Social Protection, National Health Institute, Regulatory Commission on Health, Superintendency of Health etc.), health care providers, scientific/professional societies, pharmaceutical companies, third party payers (insurance companies), patients and human rights activists were identified and participated in identifying barriers and facilitators to the Guideline implementation. 

### V. Identify barriers and facilitators to implementation of equity-focused recommendations

The guideline panel should engage in a collaborative process with the actors who were identified in the actor mapping process to recognize the barriers and facilitators for guideline implementation. Special attention should be paid to those barriers and facilitators that can prevent or cause IGI’s (Lorenc *et al.* 2013) and influence the success of achieving equity-focused goals. Identification of barriers and facilitators can be supported through the use of a formal tool, such as the ‘PEST’ analysis, where Political, Economic, Societal and Technological factors are considered (Burt *et al.* 2006). It is highly relevant to separately identify barriers and facilitators for disadvantaged groups and the institutions in which guidelines are going to be implemented. Also, and dependent upon the institutions for which the guidelines are relevant, the barriers may affect health inequities differently and so specific contextual factors related to different institutions may require specific consideration in the guidelines.

In the CCase, providers and third party payers identified some barriers related to the types of health plans. The health care system in Colombia provides different kinds of benefits depending on the plan with which the person is affiliated: contributive (workers and enterprises contribute) or subsidized (by the government). The subsidized plan provides fewer benefits and is usually of lower quality, compared with the contributive plan. As well, the subsidized plan is that with which disadvantaged populations are most often affiliated. Some interventions that are evident in the contributive plan and that are recommended by guidelines are not covered or accessible for those affiliated with the subsidized plan (e.g. epidurals analgesia, options to have a caesarean section, etc.). The issues related to subsidized plans are exacerbated by a lack of healthcare personnel and technological resources in remote and/or under resourced areas where those who experience disadvantage are most likely to live, as well as healthcare costs (copayments).

### VI. Design strategies to overcome the identified barriers

Once barriers and facilitators for guideline implementation of equity-focused recommendations are identified, the guideline implementation team determines the appropriate interventions to achieve the guideline implementation goals. First, the guideline panel develops an ‘equity-strategy’ aimed at overcoming the identified barriers and leveraging facilitators for equity-focused recommendations and thereby reaching disadvantaged groups. Next, the selection of interventions that are to be tailored to the setting in which the guideline will be implemented should be based on literature review, contextual factors, and expert opinion. The latter task may be beyond the scope of guideline developers, hence the main responsibility for intervention selection may be that of the policymaker/programme developers who are implementing the recommendations. The equity-strategy should be oriented towards reduction of the potential for IGIs and this consideration may be incorporated into the guideline implementation plan. Additionally, an iterative approach to equity-strategy development can be used for continuous improvement (Tugwell *et al.* 1985; Welch *et al.* 2008). Plans can be made so that, during guideline implementation, monitoring of impact and uptake can be used to modify interventions and to identify and address the barriers that affect the adoption of guideline recommendations.

In the CCase, some strategies to overcome the recognized barriers were identified and recommended within the guideline:

- Establish a specific plan to identify and reach pregnant women at high risk for food insecurity and hunger; in the CCase example, the plan was framed using a human rights perspective, and the aim to achieve, the Millennium Development Goals.
- Prior to initiation of the implementation process, it was recommended that policy makers issue a national policy and make an explicit agreement with third party payers and providers to ensure the adoption of the Guideline.
- Recommendations were made for economic incentives to encourage care providers to prioritize pregnant women as those specifically at risk of food insecurity and hunger.

In the CCase these recommendations were included in the guideline and this implementation step was the responsibility of the Ministry of Health.

### VII. Define resources and incentives

The guideline panel should consider financial and non-financial incentives as well as the resources required to facilitate the adoption of the specific equity strategies proposed in the guidelines. These incentives and resources must be identified (e.g. provision of a pregnancy planning service to remote communities), measured (e.g. number of health workers and transportation requirements to reach remote clinics) and valued (e.g. monetary costs). The GRADE EtD frameworks provide tools to consider feasibility of guideline implementation (Alonso-Coello *et al.* 2016).

In the CCase, the panel suggested that mechanisms for institutional and professional incentives for implementation of the guideline with the equity recommendations be integrated within the framework of the general system of health care quality and the institutional systems of incentives. The panel suggested including these economic incentive schemes as part of the economic analysis, as well as carrying out a budget impact analysis prior to implementation and during the guideline follow up. As in the previous step, this implementation step was the responsibility of the Ministry of Health.

### VIII. Design a communication strategy with an equity focus

The guideline panel should develop a communication strategy that aims to ensure that the newly developed guideline is disseminated to those who will use and implement its recommendations. Diffusion and dissemination strategies for healthcare professionals must emphasize equity goals as well as the importance of prioritizing disadvantaged groups during the guideline implementation process (Freiler *et al.* 2013).

Patient versions of guidelines must be written in a way that considers literacy barriers that may be experienced by the target user groups. Information, Education, Communication (IEC) strategies must be tailored to the range of demographic, structural, and cultural features of different population subgroups. The IEC strategies may be employed using verbal methods (e.g. healthcare providers talking with their patients) or through media using methods to foster literacy skills and engagement with information (e.g. television, cell phones, radio, social media and/or with pictures, figures/graphs and stories with minimal text). A good example of different IEC strategies are the patient and consumer guidelines developed by the Agency for Health Research and Quality (Agency for Healthcare Research and Quality, 2014).

In the CCase, a patient version of the guideline was written using plain language and in a shortened format (easy-to-understand); this was available to patients and to health professionals responsible for implementation of the guideline. Messages directed at empowering pregnant women and the people around them to demand and fulfill their rights were also included in this version of the guideline.

### IX. Define the monitoring and evaluation strategies

Strategies for monitoring and evaluating how guideline recommendations impact health inequities must consider measuring equity focused indicators and/or indices (World Health Organization 2008).

In the CCase, the panel suggested developing monitoring indicators with socioeconomic status or education as stratification variables to ensure that health disparities are monitored. Estimates for the reporting of disaggregated measures by providers and third party payers was suggested by the panel, to facilitate identification of those providers and third party payers who may be nonadherent. Indicators were proposed to monitor the implementation of guidelines with disadvantaged populations; e.g., the incidence of pregnant women who experience malnutrition in relation to the total number of women in prenatal care per year, by third payer; and the stillbirths ratio (the sum of the total number of foetal deaths in relation to the total number of live births), as a proxy outcome of maternal malnutrition. As in the previous steps, this step was also under responsibility of the Ministry of Health.

### Methodological and contextual challenges

The intrinsic challenges of incorporating equity into guideline implementation include more work for guideline developers and the possibility for greater costs associated with actual guideline implementation. It also requires that some members of the guideline development panel have expertise on how to design equity-focused implementation and evaluation plans, and that includes engaging representatives of disadvantaged groups in the guideline planning process (Akl *et al.* 2017).

In addition, the lack of validated tools to guide and monitor guideline implementation in general and from an equity perspective in particular is a challenge for both guideline developers and for policy maker/programme developers. As equity considerations may conflict with efficiency goals for resource allocation, wider analysis considering the ethical dilemmas that may be posed by implementing (and not implementing) equity-focused interventions as well as cost effectiveness and budget impact analysis of the proposed equity-focused guideline strategy should be done.

An extrinsic factor that could limit implementation of an equity-focused guideline is that the social commitments of policymakers/programme developers to reduce social inequities may vary over time and therefore it may influence resources that are made available for the development of equity-focused strategies. In addition to the potential for external limitations on guideline development, guideline developers should ideally engage in addressing equity issues throughout the whole process of guideline development and support monitoring of the impacts of their recommendations to ensure they are not worsening health inequities (Smith and Katikireddi, 2013).

### Research agenda

Aside from the Colombian guideline implementation case study described in this article, the steps providing guidance for consideration of equity in the guideline implementation process have not yet been used for the implementation of other guidelines. Further research on implementation of guidelines is essential to assess the feasibility, acceptability, cost and impact of the nine steps described here, as well as any instances in which the guideline content or processes are modified.

## Conclusion

This article presents a case study that highlights nine steps for guideline developers to explicitly consider equity issues during guideline implementation planning. The implementation of equity-focused guidelines requires commitments from political and other key decision makers as well as strong interdisciplinary and trans-sectorial collaboration. Guideline developers must set priorities and support initiatives that actively promote equity in the planning, execution and evaluation of equity-focused guideline implementation. Inclusion of guideline stakeholders throughout the implementation planning process should include representatives from disadvantaged groups, and are crucial for planning guideline implementation strategies.
